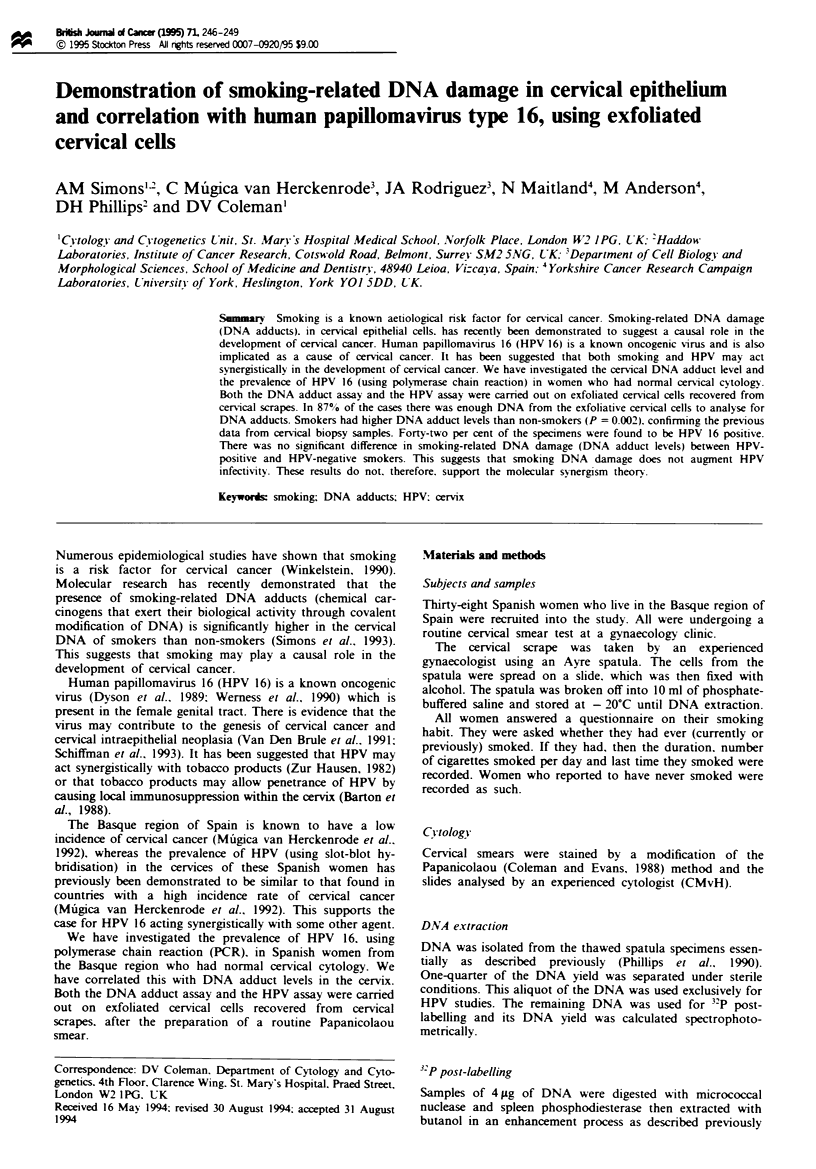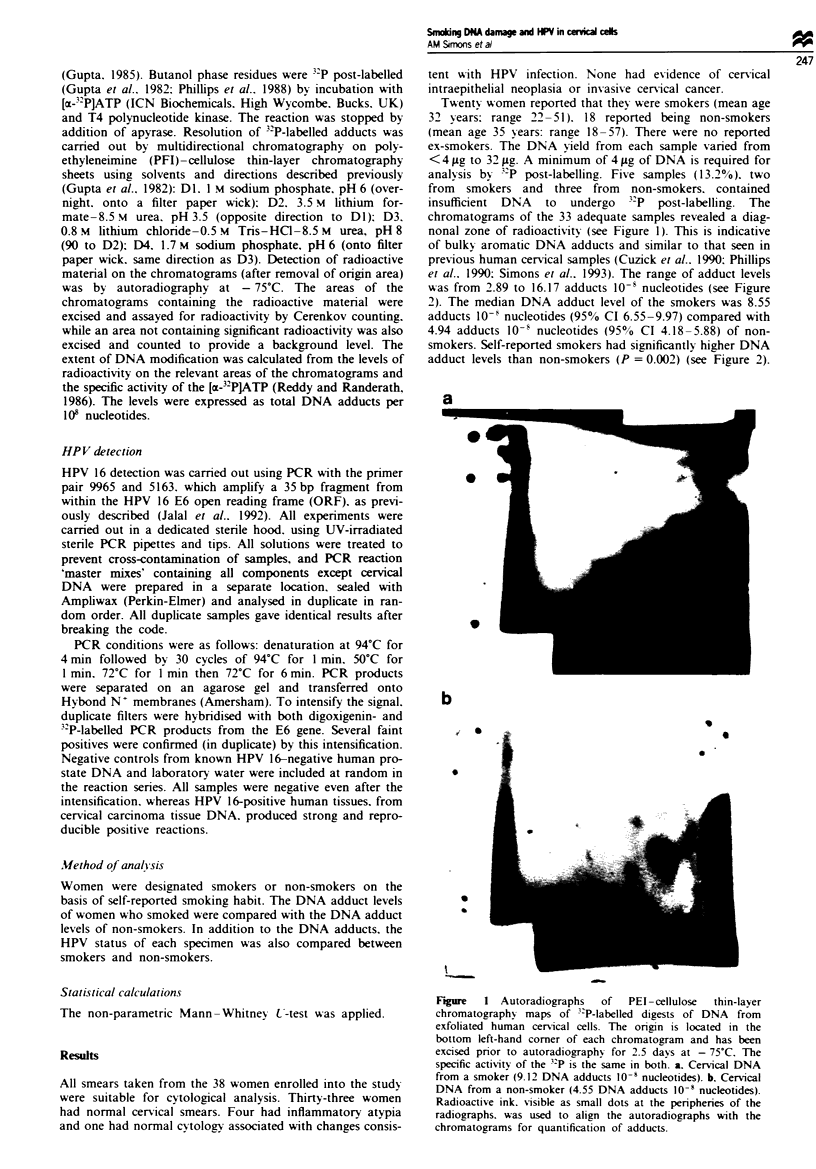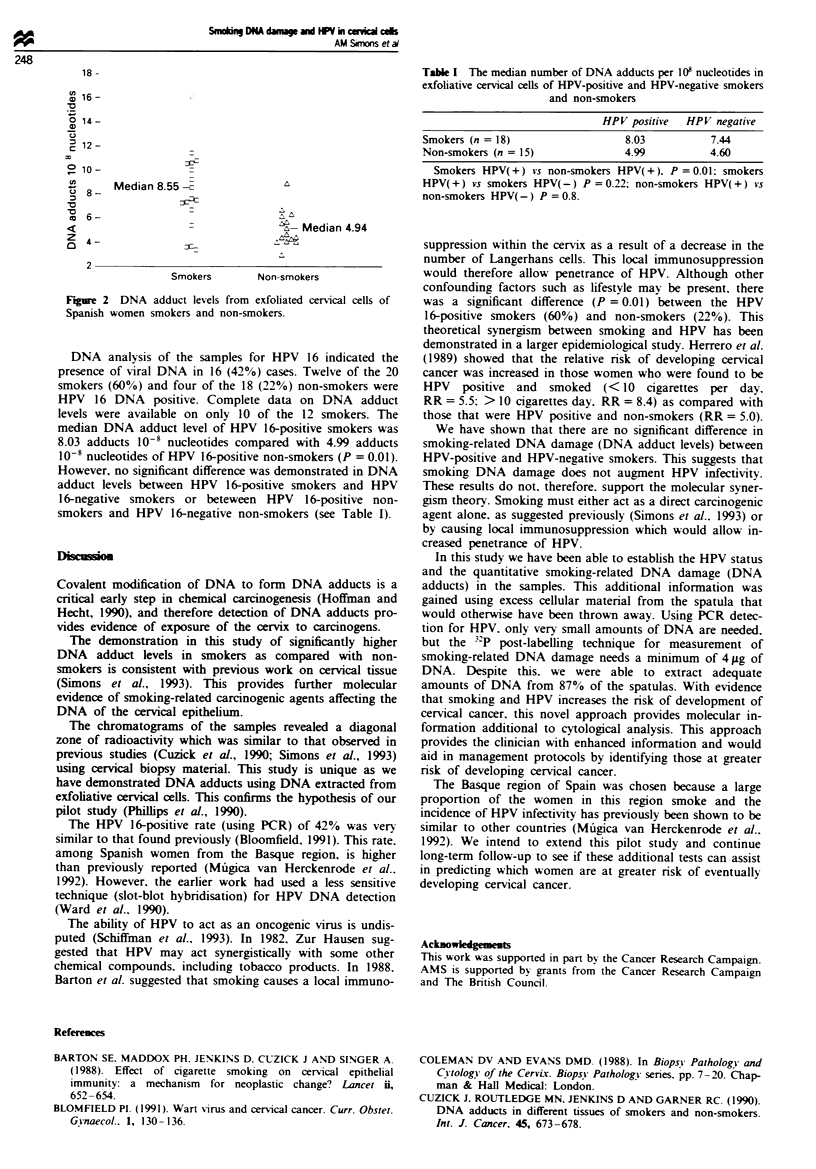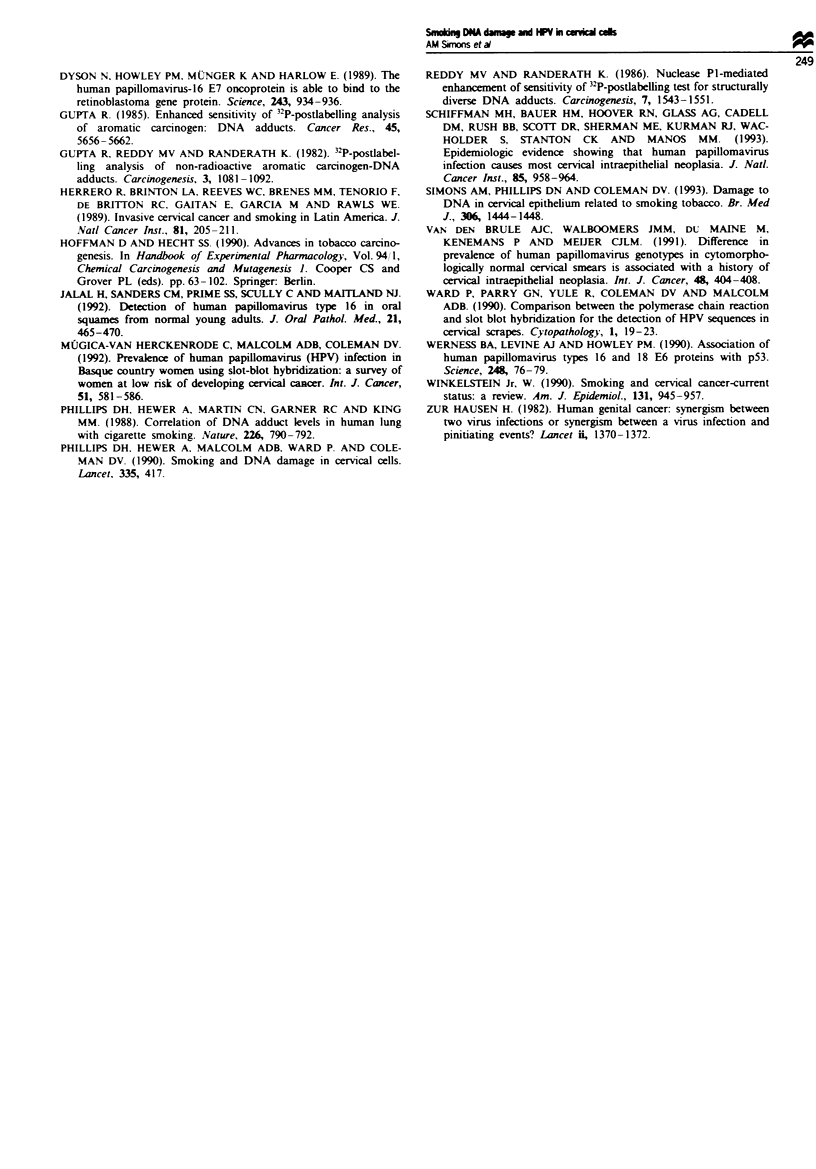# Demonstration of smoking-related DNA damage in cervical epithelium and correlation with human papillomavirus type 16, using exfoliated cervical cells.

**DOI:** 10.1038/bjc.1995.51

**Published:** 1995-02

**Authors:** A. M. Simons, C. Múgica van Herckenrode, J. A. Rodriguez, N. Maitland, M. Anderson, D. H. Phillips, D. V. Coleman

**Affiliations:** Cytology and Cytogenetics Unit, St. Mary's Hospital Medical School, London, UK.

## Abstract

**Images:**


British Journal of Cancer (1995) 71, 246-249

© 1995 Stockton Press All rights reserved 0007-0920/95 $9.00

### Demonstration of smoking-related DNA damage in cervical epithelium and correlation with human papillomavirus type 16, using exfoliated cervical cells

AM Simons[1,2], C Múgica van Herckenrode[3], JA Rodriguez[3], N Maitland[4], M Anderson[4], DH Phillips[2] and DV Coleman[1]

[1]*Cytology and Cytogenetics Unit, St. Mary's Hospital Medical School, Norfolk Place, London W2 1PG, UK;* [2]*Haddow Laboratories, Institute of Cancer Research, Cotswold Road, Belmont, Surrey SM2 5NG, UK;* [3]*Department of Cell Biology and Morphological Sciences, School of Medicine and Dentistry, 48940 Leioa, Vizcaya, Spain;* [4]*Yorkshire Cancer Research Campaign Laboratories, University of York, Heslington, York YO1 5DD, UK.*

**Summary** Smoking is a known aetiological risk factor for cervical cancer. Smoking-related DNA damage (DNA adducts), in cervical epithelial cells, has recently been demonstrated to suggest a causal role in the development of cervical cancer. Human papillomavirus 16 (HPV 16) is a known oncogenic virus and is also implicated as a cause of cervical cancer. It has been suggested that both smoking and HPV may act synergistically in the development of cervical cancer. We have investigated the cervical DNA adduct level and the prevalence of HPV 16 (using polymerase chain reaction) in women who had normal cervical cytology. Both the DNA adduct assay and the HPV assay were carried out on exfoliated cervical cells recovered from cervical scrapes. In 87% of the cases there was enough DNA from the exfoliative cervical cells to analyse for DNA adducts. Smokers had higher DNA adduct levels than non-smokers ($P = 0.002$), confirming the previous data from cervical biopsy samples. Forty-two per cent of the specimens were found to be HPV 16 positive. There was no significant difference in smoking-related DNA damage (DNA adduct levels) between HPV-positive and HPV-negative smokers. This suggests that smoking DNA damage does not augment HPV infectivity. These results do not, therefore, support the molecular synergism theory.

**Keywords:** smoking; DNA adducts; HPV; cervix

Numerous epidemiological studies have shown that smoking is a risk factor for cervical cancer (Winkelstein, 1990). Molecular research has recently demonstrated that the presence of smoking-related DNA adducts (chemical carcinogens that exert their biological activity through covalent modification of DNA) is significantly higher in the cervical DNA of smokers than non-smokers (Simons *et al*., 1993). This suggests that smoking may play a causal role in the development of cervical cancer.

Human papillomavirus 16 (HPV 16) is a known oncogenic virus (Dyson *et al*., 1989; Werness *et al*., 1990) which is present in the female genital tract. There is evidence that the virus may contribute to the genesis of cervical cancer and cervical intraepithelial neoplasia (Van Den Brule *et al*., 1991; Schiffman *et al*., 1993). It has been suggested that HPV may act synergistically with tobacco products (Zur Hausen, 1982) or that tobacco products may allow penetrance of HPV by causing local immunosuppression within the cervix (Barton *et al*., 1988).

The Basque region of Spain is known to have a low incidence of cervical cancer (Múgica van Herckenrode *et al*., 1992), whereas the prevalence of HPV (using slot-blot hybridisation) in the cervices of these Spanish women has previously been demonstrated to be similar to that found in countries with a high incidence rate of cervical cancer (Múgica van Herckenrode *et al*., 1992). This supports the case for HPV 16 acting synergistically with some other agent.

We have investigated the prevalence of HPV 16, using polymerase chain reaction (PCR), in Spanish women from the Basque region who had normal cervical cytology. We have correlated this with DNA adduct levels in the cervix. Both the DNA adduct assay and the HPV assay were carried out on exfoliated cervical cells recovered from cervical scrapes, after the preparation of a routine Papanicolaou smear.

Correspondence: DV Coleman, Department of Cytology and Cytogenetics, 4th Floor, Clarence Wing, St. Mary's Hospital, Praed Street, London W2 1PG, UK
Received 16 May 1994; revised 30 August 1994; accepted 31 August 1994

### Materials and methods

### *Subjects and samples*

Thirty-eight Spanish women who live in the Basque region of Spain were recruited into the study. All were undergoing a routine cervical smear test at a gynaecology clinic.

The cervical scrape was taken by an experienced gynaecologist using an Ayre spatula. The cells from the spatula were spread on a slide, which was then fixed with alcohol. The spatula was broken off into 10 ml of phosphate-buffered saline and stored at −20°C until DNA extraction.

All women answered a questionnaire on their smoking habit. They were asked whether they had ever (currently or previously) smoked. If they had, then the duration, number of cigarettes smoked per day and last time they smoked were recorded. Women who reported to have never smoked were recorded as such.

### *Cytology*

Cervical smears were stained by a modification of the Papanicolaou (Coleman and Evans, 1988) method and the slides analysed by an experienced cytologist (CMvH).

### *DNA extraction*

DNA was isolated from the thawed spatula specimens essentially as described previously (Phillips *et al*., 1990). One-quarter of the DNA yield was separated under sterile conditions. This aliquot of the DNA was used exclusively for HPV studies. The remaining DNA was used for $^{32}$P post-labelling and its DNA yield was calculated spectrophotometrically.

### *$^{32}$P post-labelling*

Samples of 4 μg of DNA were digested with micrococcal nuclease and spleen phosphodiesterase then extracted with butanol in an enhancement process as described previously

(Gupta, 1985). Butanol phase residues were $^{32}P$ post-labelled (Gupta *et al.*, 1982; Phillips *et al.*, 1988) by incubation with [α-$^{32}P$]ATP (ICN Biochemicals, High Wycombe, Bucks, UK) and T4 polynucleotide kinase. The reaction was stopped by addition of apyrase. Resolution of $^{32}P$-labelled adducts was carried out by multidirectional chromatography on polyethyleneimine (PFI)-cellulose thin-layer chromatography sheets using solvents and directions described previously (Gupta *et al.*, 1982): D1, 1 M sodium phosphate, pH 6 (overnight, onto a filter paper wick); D2, 3.5 M lithium formate-8.5 M urea, pH 3.5 (opposite direction to D1); D3, 0.8 M lithium chloride-0.5 M Tris-HCl-8.5 M urea, pH 8 (90 to D2); D4, 1.7 M sodium phosphate, pH 6 (onto filter paper wick, same direction as D3). Detection of radioactive material on the chromatograms (after removal of origin area) was by autoradiography at − 75°C. The areas of the chromatograms containing the radioactive material were excised and assayed for radioactivity by Cerenkov counting, while an area not containing significant radioactivity was also excised and counted to provide a background level. The extent of DNA modification was calculated from the levels of radioactivity on the relevant areas of the chromatograms and the specific activity of the [α-$^{32}P$]ATP (Reddy and Randerath, 1986). The levels were expressed as total DNA adducts per $10^8$ nucleotides.

### *HPV detection*

HPV 16 detection was carried out using PCR with the primer pair 9965 and 5163, which amplify a 35 bp fragment from within the HPV 16 E6 open reading frame (ORF), as previously described (Jalal *et al.*, 1992). All experiments were carried out in a dedicated sterile hood, using UV-irradiated sterile PCR pipettes and tips. All solutions were treated to prevent cross-contamination of samples, and PCR reaction 'master mixes' containing all components except cervical DNA were prepared in a separate location, sealed with Ampliwax (Perkin-Elmer) and analysed in duplicate in random order. All duplicate samples gave identical results after breaking the code.

PCR conditions were as follows: denaturation at 94°C for 4 min followed by 30 cycles of 94°C for 1 min, 50°C for 1 min, 72°C for 1 min then 72°C for 6 min. PCR products were separated on an agarose gel and transferred onto Hybond $N^+$ membranes (Amersham). To intensify the signal, duplicate filters were hybridised with both digoxigenin- and $^{32}P$-labelled PCR products from the E6 gene. Several faint positives were confirmed (in duplicate) by this intensification. Negative controls from known HPV 16-negative human prostate DNA and laboratory water were included at random in the reaction series. All samples were negative even after the intensification, whereas HPV 16-positive human tissues, from cervical carcinoma tissue DNA, produced strong and reproducible positive reactions.

### *Method of analysis*

Women were designated smokers or non-smokers on the basis of self-reported smoking habit. The DNA adduct levels of women who smoked were compared with the DNA adduct levels of non-smokers. In addition to the DNA adducts, the HPV status of each specimen was also compared between smokers and non-smokers.

### *Statistical calculations*

The non-parametric Mann-Whitney *U*-test was applied.

### Results

All smears taken from the 38 women enrolled into the study were suitable for cytological analysis. Thirty-three women had normal cervical smears. Four had inflammatory atypia and one had normal cytology associated with changes consistent with HPV infection. None had evidence of cervical intraepithelial neoplasia or invasive cervical cancer.

Twenty women reported that they were smokers (mean age 32 years; range 22-51), 18 reported being non-smokers (mean age 35 years; range 18-57). There were no reported ex-smokers. The DNA yield from each sample varied from <4 μg to 32 μg. A minimum of 4 μg of DNA is required for analysis by $^{32}P$ post-labelling. Five samples (13.2%), two from smokers and three from non-smokers, contained insufficient DNA to undergo $^{32}P$ post-labelling. The chromatograms of the 33 adequate samples revealed a diagnonal zone of radioactivity (see Figure 1). This is indicative of bulky aromatic DNA adducts and similar to that seen in previous human cervical samples (Cuzick *et al.*, 1990; Phillips *et al.*, 1990; Simons *et al.*, 1993). The range of adduct levels was from 2.89 to 16.17 adducts $10^{-8}$ nucleotides (see Figure 2). The median DNA adduct level of the smokers was 8.55 adducts $10^{-8}$ nucleotides (95% CI 6.55-9.97) compared with 4.94 adducts $10^{-8}$ nucleotides (95% CI 4.18-5.88) of non-smokers. Self-reported smokers had significantly higher DNA adduct levels than non-smokers ($P = 0.002$) (see Figure 2).

**Figure 1** Autoradiographs of PEI-cellulose thin-layer chromatography maps of $^{32}P$-labelled digests of DNA from exfoliated human cervical cells. The origin is located in the bottom left-hand corner of each chromatogram and has been excised prior to autoradiography for 2.5 days at − 75°C. The specific activity of the $^{32}P$ is the same in both. **a**, Cervical DNA from a smoker (9.12 DNA adducts $10^{-8}$ nucleotides). **b**, Cervical DNA from a non-smoker (4.55 DNA adducts $10^{-8}$ nucleotides). Radioactive ink, visible as small dots at the peripheries of the radiographs, was used to align the autoradiographs with the chromatograms for quantification of adducts.

**Figure 2** DNA adduct levels from exfoliated cervical cells of Spanish women smokers and non-smokers.

DNA analysis of the samples for HPV 16 indicated the presence of viral DNA in 16 (42%) cases. Twelve of the 20 smokers (60%) and four of the 18 (22%) non-smokers were HPV 16 DNA positive. Complete data on DNA adduct levels were available on only 10 of the 12 smokers. The median DNA adduct level of HPV 16-positive smokers was 8.03 adducts $10^{-8}$ nucleotides compared with 4.99 adducts $10^{-8}$ nucleotides of HPV 16-positive non-smokers ($P = 0.01$). However, no significant difference was demonstrated in DNA adduct levels between HPV 16-positive smokers and HPV 16-negative smokers or beteween HPV 16-positive non-smokers and HPV 16-negative non-smokers (see Table I).

**Table I** The median number of DNA adducts per $10^8$ nucleotides in exfoliative cervical cells of HPV-positive and HPV-negative smokers and non-smokers

| | *HPV positive* | *HPV negative* |
|---|---|---|
| Smokers ($n = 18$) | 8.03 | 7.44 |
| Non-smokers ($n = 15$) | 4.99 | 4.60 |

Smokers HPV(+) *vs* non-smokers HPV(+), $P = 0.01$; smokers HPV(+) *vs* smokers HPV(−) $P = 0.22$; non-smokers HPV(+) *vs* non-smokers HPV(−) $P = 0.8$.

### Discussion

Covalent modification of DNA to form DNA adducts is a critical early step in chemical carcinogenesis (Hoffman and Hecht, 1990), and therefore detection of DNA adducts provides evidence of exposure of the cervix to carcinogens.

The demonstration in this study of significantly higher DNA adduct levels in smokers as compared with non-smokers is consistent with previous work on cervical tissue (Simons *et al.*, 1993). This provides further molecular evidence of smoking-related carcinogenic agents affecting the DNA of the cervical epithelium.

The chromatograms of the samples revealed a diagonal zone of radioactivity which was similar to that observed in previous studies (Cuzick *et al.*, 1990; Simons *et al.*, 1993) using cervical biopsy material. This study is unique as we have demonstrated DNA adducts using DNA extracted from exfoliative cervical cells. This confirms the hypothesis of our pilot study (Phillips *et al.*, 1990).

The HPV 16-positive rate (using PCR) of 42% was very similar to that found previously (Bloomfield, 1991). This rate, among Spanish women from the Basque region, is higher than previously reported (Múgica van Herckenrode *et al.*, 1992). However, the earlier work had used a less sensitive technique (slot-blot hybridisation) for HPV DNA detection (Ward *et al.*, 1990).

The ability of HPV to act as an oncogenic virus is undisputed (Schiffman *et al.*, 1993). In 1982, Zur Hausen suggested that HPV may act synergistically with some other chemical compounds, including tobacco products. In 1988, Barton *et al.* suggested that smoking causes a local immunosuppression within the cervix as a result of a decrease in the number of Langerhans cells. This local immunosuppression would therefore allow penetrance of HPV. Although other confounding factors such as lifestyle may be present, there was a significant difference ($P = 0.01$) between the HPV 16-positive smokers (60%) and non-smokers (22%). This theoretical synergism between smoking and HPV has been demonstrated in a larger epidemiological study. Herrero *et al.* (1989) showed that the relative risk of developing cervical cancer was increased in those women who were found to be HPV positive and smoked (<10 cigarettes per day, RR = 5.5; >10 cigarettes day, RR = 8.4) as compared with those that were HPV positive and non-smokers (RR = 5.0).

We have shown that there are no significant difference in smoking-related DNA damage (DNA adduct levels) between HPV-positive and HPV-negative smokers. This suggests that smoking DNA damage does not augment HPV infectivity. These results do not, therefore, support the molecular synergism theory. Smoking must either act as a direct carcinogenic agent alone, as suggested previously (Simons *et al.*, 1993) or by causing local immunosuppression which would allow increased penetrance of HPV.

In this study we have been able to establish the HPV status and the quantitative smoking-related DNA damage (DNA adducts) in the samples. This additional information was gained using excess cellular material from the spatula that would otherwise have been thrown away. Using PCR detection for HPV, only very small amounts of DNA are needed, but the $^{32}P$ post-labelling technique for measurement of smoking-related DNA damage needs a minimum of 4 μg of DNA. Despite this, we were able to extract adequate amounts of DNA from 87% of the spatulas. With evidence that smoking and HPV increases the risk of development of cervical cancer, this novel approach provides molecular information additional to cytological analysis. This approach provides the clinician with enhanced information and would aid in management protocols by identifying those at greater risk of developing cervical cancer.

The Basque region of Spain was chosen because a large proportion of the women in this region smoke and the incidence of HPV infectivity has previously been shown to be similar to other countries (Múgica van Herckenrode *et al.*, 1992). We intend to extend this pilot study and continue long-term follow-up to see if these additional tests can assist in predicting which women are at greater risk of eventually developing cervical cancer.

**Acknowledgements**

This work was supported in part by the Cancer Research Campaign. AMS is supported by grants from the Cancer Research Campaign and The British Council.

### References

BARTON SE, MADDOX PH, JENKINS D, CUZICK J AND SINGER A. (1988). Effect of cigarette smoking on cervical epithelial immunity: a mechanism for neoplastic change? *Lancet* **ii**, 652–654.

BLOMFIELD PI. (1991). Wart virus and cervical cancer. *Curr. Obstet. Gynaecol.*, **1**, 130–136.

COLEMAN DV AND EVANS DMD. (1988). In *Biopsy Pathology and Cytology of the Cervix. Biopsy Pathology* series, pp. 7–20. Chapman & Hall Medical: London.

CUZICK J, ROUTLEDGE MN, JENKINS D AND GARNER RC. (1990). DNA adducts in different tissues of smokers and non-smokers. *Int. J. Cancer*, **45**, 673–678.

DYSON N, HOWLEY PM, MÜNGER K AND HARLOW E. (1989). The human papillomavirus-16 E7 oncoprotein is able to bind to the retinoblastoma gene protein. *Science*, **243**, 934-936.

GUPTA R. (1985). Enhanced sensitivity of $^{32}$P-postlabelling analysis of aromatic carcinogen: DNA adducts. *Cancer Res.*, **45**, 5656-5662.

GUPTA R, REDDY MV AND RANDERATH K. (1982). $^{32}$P-postlabelling analysis of non-radioactive aromatic carcinogen-DNA adducts. *Carcinogenesis*, **3**, 1081-1092.

HERRERO R, BRINTON LA, REEVES WC, BRENES MM, TENORIO F, DE BRITTON RC, GAITAN E, GARCIA M AND RAWLS WE. (1989). Invasive cervical cancer and smoking in Latin America. *J. Natl Cancer Inst.*, **81**, 205-211.

HOFFMAN D AND HECHT SS. (1990). Advances in tobacco carcinogenesis. In *Handbook of Experimental Pharmacology*, Vol. 94 1, *Chemical Carcinogenesis and Mutagenesis 1*. Cooper CS and Grover PL (eds). pp. 63-102. Springer: Berlin.

JALAL H, SANDERS CM, PRIME SS, SCULLY C AND MAITLAND NJ. (1992). Detection of human papillomavirus type 16 in oral squames from normal young adults. *J. Oral Pathol. Med.*, **21**, 465-470.

MÚGICA-VAN HERCKENRODE C, MALCOLM ADB, COLEMAN DV. (1992). Prevalence of human papillomavirus (HPV) infection in Basque country women using slot-blot hybridization: a survey of women at low risk of developing cervical cancer. *Int. J. Cancer*, **51**, 581-586.

PHILLIPS DH, HEWER A, MARTIN CN, GARNER RC AND KING MM. (1988). Correlation of DNA adduct levels in human lung with cigarette smoking. *Nature*, **226**, 790-792.

PHILLIPS DH, HEWER A, MALCOLM ADB, WARD P AND COLEMAN DV. (1990). Smoking and DNA damage in cervical cells. *Lancet*, **335**, 417.

REDDY MV AND RANDERATH K. (1986). Nuclease P1-mediated enhancement of sensitivity of $^{32}$P-postlabelling test for structurally diverse DNA adducts. *Carcinogenesis*, **7**, 1543-1551.

SCHIFFMAN MH, BAUER HM, HOOVER RN, GLASS AG, CADELL DM, RUSH BB, SCOTT DR, SHERMAN ME, KURMAN RJ, WACHOLDER S, STANTON CK AND MANOS MM. (1993). Epidemiologic evidence showing that human papillomavirus infection causes most cervical intraepithelial neoplasia. *J. Natl. Cancer Inst.*, **85**, 958-964.

SIMONS AM, PHILLIPS DN AND COLEMAN DV. (1993). Damage to DNA in cervical epithelium related to smoking tobacco. *Br. Med J.*, **306**, 1444-1448.

VAN DEN BRULE AJC, WALBOOMERS JMM, DU MAINE M, KENEMANS P AND MEIJER CJLM. (1991). Difference in prevalence of human papillomavirus genotypes in cytomorphologically normal cervical smears is associated with a history of cervical intraepithelial neoplasia. *Int. J. Cancer*, **48**, 404-408.

WARD P, PARRY GN, YULE R, COLEMAN DV AND MALCOLM ADB. (1990). Comparison between the polymerase chain reaction and slot blot hybridization for the detection of HPV sequences in cervical scrapes. *Cytopathology*, **1**, 19-23.

WERNESS BA, LEVINE AJ AND HOWLEY PM. (1990). Association of human papillomavirus types 16 and 18 E6 proteins with p53. *Science*, **248**, 76-79.

WINKELSTEIN Jr. W. (1990). Smoking and cervical cancer-current status: a review. *Am. J. Epidemiol.*, **131**, 945-957.

ZUR HAUSEN H. (1982). Human genital cancer: synergism between two virus infections or synergism between a virus infection and pinitiating events? *Lancet* **ii**, 1370-1372.