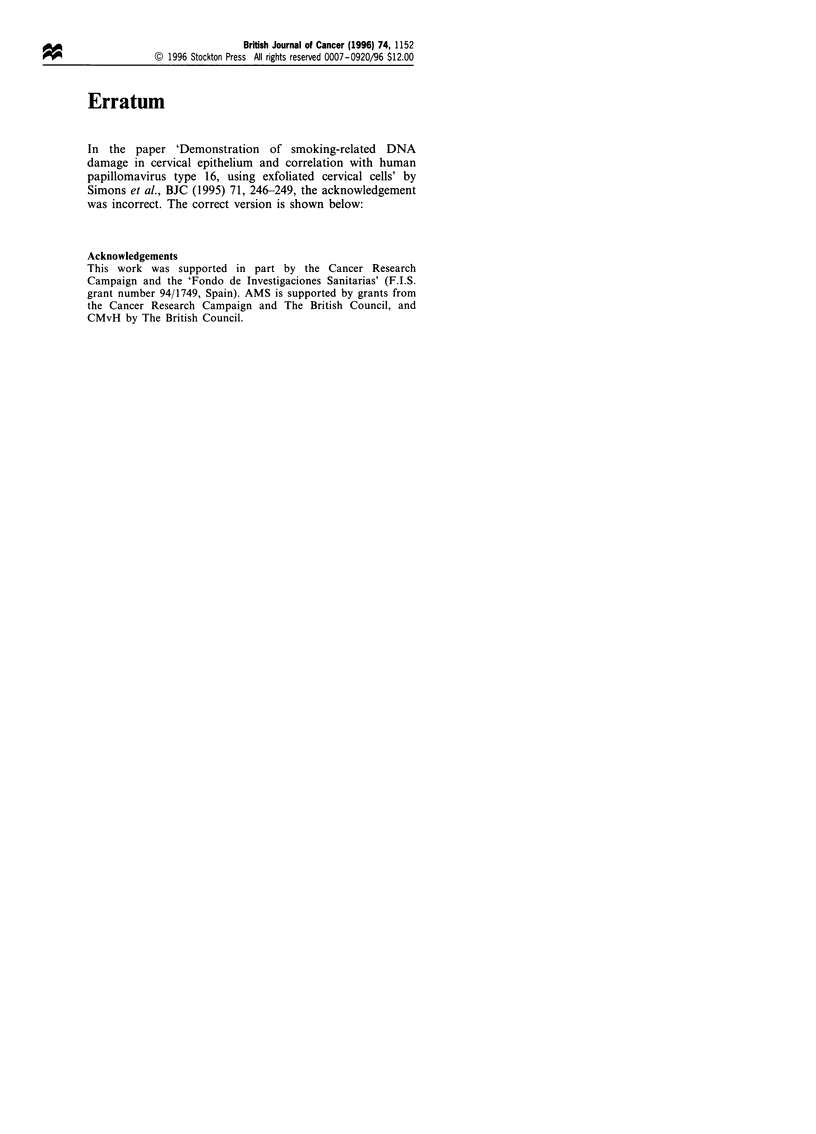# Erratum

**Published:** 1996-10

**Authors:** 


British Journal of Cancer (1996) 74, 1152
© 1996 Stockton Press All rights reserved 0007-0920/96 $12.00

### Erratum

In the paper 'Demonstration of smoking-related DNA damage in cervical epithelium and correlation with human papillomavirus type 16, using exfoliated cervical cells' by Simons *et al.*, BJC (1995) 71, 246–249, the acknowledgement was incorrect. The correct version is shown below:

**Acknowledgements**
This work was supported in part by the Cancer Research Campaign and the 'Fondo de Investigaciones Sanitarias' (F.I.S. grant number 94/1749, Spain). AMS is supported by grants from the Cancer Research Campaign and The British Council, and CMvH by The British Council.